# Augmented CD133 expression in distal margin correlates with poor prognosis in colorectal cancer

**DOI:** 10.1111/jcmm.14284

**Published:** 2019-04-04

**Authors:** Tapas Pradhan, Krishnanand Padmanabhan, Manu Prasad, K. Chandramohan, S. Asha Nair

**Affiliations:** ^1^ Cancer Research Program 4 Rajiv Gandhi Centre for Biotechnology Trivandrum Kerala India; ^2^ Department of surgical oncology Regional Cancer Centre Trivandrum Kerala India

**Keywords:** cancer stem cell marker, colorectal cancer, disease recurrence, distal margin, prognosis

## Abstract

Pathological assessment of excised tumour and surgical margins in colorectal cancer (CRC) play crucial role in prognosis after surgery. Molecular assessment of margins could be more sensitive and informative than conventional histopathological analysis. Considering this view, we evaluated the distal surgical margins for expression of cancer stem cell (CSC) markers. Cellular and molecular assessment of normal, tumour and distal margin tissues were performed by flow cytometry, real‐time q‐PCR and immuno‐histochemical analysis for CRC patients after tumour excision. CRC patients were evaluated for expression of CSC markers in their normal, tumour and distal tissues. Flow cytometry assay revealed CD133 and CD44 enriched cells in distal margin and tumour compared to normal colorectal tissues, which was further confirmed by immunohistochemistry. Most importantly, immunohistochemistry also revealed the enrichment of CSC markers expression in pathologically negative distal margins. Patients with distal margin enriched for CD133 expression showed an increased recurrence rate and decreased disease‐free survival. This study proposes that although distal margin seems to be tumour free in conventional histopathological analysis, it could harbour cells enriched for CSC markers. Further CD133 could be a promising molecule to be used in molecular pathology for disease prognosis after surgery in CRC patients.

## 1 INTRODUCTION

Colorectal cancer (CRC) is the third most commonly occurring cancer globally regardless of sex. In the year 2016, the United States solely had 134,490 new CRC cases (NCI, 2016). Recent reports have shown that there is an increase in CRC incidence in young population in many resource‐constrained countries including India^1^ and the 5‐year survival rate in India is below 40%, which is one of the lowest compared to western countries.^2^ CRC treatment and management have indeed evolved over a couple of decades due to improved screening and therapeutic modalities. This paradigm shift in CRC regimen has been possible because of extensive knowledge about the pivotal molecular players and pathways involved in the disease, which has finally aided in augmentation of potent anti‐tumour therapeutics. Despite these significant developments, disease relapse and distant metastasis remain as major challenges in CRC.^3^ It is known that around 20% to 40% of patients undergoing curative resection for CRC develop loco‐regional recurrence or distant metastasis.^4^ Although chemoradiotherapy has radically enhanced the treatment efficacy in CRC, still surgery remains the conclusive treatment regimen given to most patients. Removal of all residual cells in and around tumour has been suggestive of an effective surgery. In CRC, excision of surgical margins along with tumour is critical for curative surgery practice, as studies have shown that positive surgical margins correlate with disease recurrence and distant metastasis.^5^, ^6^ Presently, biopsies are evaluated visually by microscopic examination of hematoxylin and eosin stained slides by a pathologist. However, molecular alterations and residual tumour cells may go unnoticed in conventional pathology. Molecular evaluation of surgical margins has been described to be more sensitive and informative compared to conventional histopathological assessment in many cancers.^7^ Currently, insights on molecular signatures of surgical margins are lacking, which otherwise could aid in defining tumour‐free resectable margin in CRC. Criteria for precise selection of distal margin in a sphincter preserving surgery need more research as distal resection length still remains a topic of debate.^8^ Molecular assessment includes many advanced and precise techniques, which enable to assess many risk parameters and could be of help in improved prognosis of patients.

Presently, there is a need for robust, reliable prognostic and predictive molecular markers in CRC, which could help oncologist to decide patient‐specific treatment regimens as well as help in better patient's follow‐up post surgery. Accumulating evidence on a sub‐population of cells in tumours, demonstrated to have high tumourigenic proficiency compared to the bulk of the tumour cells and were designated as cancer stem cells (CSCs). CSCs are reported to be the possible culprits behind therapy resistance^9^, ^10^ and also responsible for disease recurrence.^11^ CD133, CD44 and Ep‐CAM have been shown to be specific for CSC cells in CRC and Ep‐CAM^high^/CD44^+^ positivity has been reported to be associated with metastasis in CRC.^12^, ^13^ CD133 has been shown to be an independent prognostic marker for lower survival rate in CRC patients.^15^ However, there are hardly any studies evaluating CSCs‐associated molecular signatures in surgical margins and their role in CRC prognosis.

CRC distal margin remains as the least resolved entity in regard to its molecular signatures and prognostic values. Our study explored distal surgical margin conjointly with tumour using established molecular markers specific for CSCs in CRC patients undergoing curative surgical resection.

## 2 MATERIAL AND METHODS

### 2.1 Patients and samples

Biopsies were collected from CRC patients undergoing curative surgery between 2013 and 2017 at the Regional Cancer Centre, Trivandrum after human ethics committee approval and sanction from Institutional Review Board. All patients gave written informed consent in accordance with the Declaration of Helsinki. Patient's details and clinical information were collected from medical records of the same institution. In this study, only adenocarcinoma was included, excluding patients exhibiting gastrointestinal tumours. Patient's demographic details are given in Table S1. Briefly, patients presented with rectal cancer had undergone a long course of radiotherapy of 50 Gy in 25 fractions followed by low anterior resection and abdominoperineal resection for upper and lower rectal cancer respectively. On the other hand, colon cancer patients went through hemicolectomy. Surgery involved removal of tumour, lymphnodes and associated margins followed by anastomosis for retaining the intestinal continuity.

The biopsies were collected from normal, tumour and distal resection margins from each patient. Biopsies were collected in HBSS (Invitrogen, USA) or in RNA stabilizing buffer (Ambion, Invitrogen, USA) immediately after surgery and transported under cold condition to the laboratory.

### 2.2 Tissue digestion and cell isolation

CRC tissues were collected after surgery in cold HBSS (Invitrogen, USA) with 2% FBS (Gibco, Invitrogen, USA) in ice and were minced into small pieces using scissors and scalpel. The minced tissues were transferred into 50 ml tube with 10 mL of DMEM media (Sigma‐Aldrich, USA) containing 200 units/mL of Collagenase type IV (Invitrogen, USA) and 2 µL of DNase (Jena Bioscience, Germany) and kept at 37°C in temperature‐controlled shaker with mild agitation for 4 hours. After that, DMEM media containing cell suspension was passed through 40 µm cell strainer (BD Bioscience, USA) and pelleted at 1600 g for 5 minutes at 4°C. RBCs were lysed by incubating cell pellet in RBC lysis solution for 5 minutes at room temperature. Cell suspension was centrifuged at 1600 *g* for 5 minutes at 4°C and supernatant was discarded. Finally, cell pellet was suspended in DMEM media supplemented with 10% serum and kept at 37°C for 3 hours for recovery.

### 2.3 Magnetic assorted cell depletion

Lymphocytes were depleted from isolated cells using CD45 Dynabeads (Invitrogen, USA), according to the manufacturer's protocol. Briefly, Dynabeads were added to cell suspension in PBS and incubated at 4°C for 30 minutes in rotary mixing. After incubation, cells with beads were placed on a magnet for 2 minutes and bound cells were discarded. Finally, supernatant was used for further analysis.

### 2.4 Immunophenotyping

Cell suspension obtained after CD45 depletion was then washed three times with HBSS buffer and pelleted by centrifugation at 1600 *g* for 5 minutes. Washed cells were suspended in 1 mL HBSS and 1 × 10^6^ cells were used for antibody staining according to the manufacturer's protocol. The details of antibodies used are provided in Table S3. After antibody staining, cells were again washed with ice‐cold HBSS and resuspended in HBSS containing 2% FBS. Cells were kept on ice till flow cytometry analysis was carried out. Flow Cytometry analysis was carried out using BD FACS ARIA II (BD Bioscience, USA).

### 2.5 RNA extraction

Total RNA was isolated from approximately 50 mg tissues (normal, tumour and distal margin) by using Trizol reagent (Invitrogen, USA) following the manufacturer's protocol. Isolated RNA quality and quantification were assessed using gel electrophoresis and Nanodrop 1000 (ThermoScientific, USA).

#### 2.5.1 mRNA sequencing and identification of mutations

Isolated RNA from normal, tumour and distal tissues collected from a CRC patient was quality checked using bioanalyzer. Whole mRNA sequencing was performed with Illumina Nextseq500 (Genotypic Technology, Bangalore, India). The NextSeq500 paired‐end raw reads were quality checked using FastQC. NextSeq 500 raw reads were processed by in‐house script for adapters and low‐quality bases trimming towards 3'‐end. TopHat‐2.0.7 and Cufflinks‐2.0.1 tools were used for the analysis. TopHat is a fast splice junction mapper for RNA‐Seq reads. It aligns RNA‐Seq reads to mammalian‐sized genomes using the ultra‐high‐throughput short read aligner Bowtie, and then analyzes the mapping results to identify splice junctions between exons. Mutations were (SNPs & INDELs) called by using SAM tools and selected only exclusive for tumour and distal samples tissues keeping normal samples reads as reference. For identification of significant variants, quality filter Q cut‐off value was set above 30 and coverage depth was kept above 20.

VENNY 2.1.0 online tool was used for comparison between tumour and distal gene mutations. Online DAVID Bioinformatics tool was used for enrichment of bioprocess and pathways (https://david.ncifcrf.gov/tools.jsp). Driver mutations were selected by comparing top 20 mutated genes in CRC from COSMIC datasets (https://cancer.sanger.ac.uk/cosmic) available online with our observed data.^16^ Further, the driver mutant genes fate was analysed by Mutation Taster bioinformatics tool.^17^

### 2.6 Quantitative real‐time‐PCR

A total of 1 µg of isolated RNA was converted to cDNA from each RNA sample using PrimerScriptcDNA conversion kit (TAKARA, Japan), according to the manufacturer's protocol. Quantitative real‐time PCR was performed with SYBR‐Green (TAKARA, Japan) based on fluorescence detection method and HT9700 detection system (AB, Life Technologies, USA). Target genes specific primers were designed using NCBI‐Primer blast tool for an amplicon size of maximum 150 bp. Analysed genes and the primers used are shown in Table S2. PCR was performed in a final volume of 5 µL containing SYBR‐Green PCR master mix, 50 ng of cDNA as template and forward and reverse primers. Relative changes in mRNA expression were obtained using ΔΔCt method keeping normal as reference sample and L19 as endogenous control using Data Assist software (AB Life Technologies, USA).

### 2.7 Immunohistochemistry

The tissues received after surgery were fixed with 4% paraformaldehyde (Sigma‐Aldrich, USA) for 16 hours at 4°C and processed with xylene and alcohol gradient followed by embedding with paraffin to make paraffin blocks. Five‐micrometre thick sections were mounted on poly L‐lysine‐coated StarFrost glass slides (Leica, Germany). The sections were deparaffinised in xylene and rehydrated with alcohol gradients. Endogenous peroxidase was blocked with 3% H_2_O_2_ in methanol for 15 minutes. The sections were immersed in prewarmed 10 mmol/L citrate buffer (pH 6.0) and kept in boiling water bath for 10 minutes. After retrieval, sections were rinsed in 1X PBS followed by BSA (3%) blocking for 30 minutes at room temperature in order to reduce nonspecific binding of antibodies. Primary antibodies anti‐EpCAM (dilution 1:100), anti‐CD44 (dilution 1:1000), anti‐CD133 (dilution 1:100) anti‐Oct4 (dilution 1:100) and anti‐β‐catenin (dilution 1:200) were added and incubated overnight at 4°C in humid chamber. Primary antibodies details are given in Table S3. After incubation, sections were washed with PBS and subsequently incubated with AB (avidin‐biotin‐peroxidase complex) (Vecta stain kit, USA) for 30 minutes. Diaminobenzidine (Sigma‐Aldrich, USA) was used as a chromogen and haematoxylin (Merck Millipore, Germany) was used as a counter stain. Sections without incubation of primary antibody served as negative control. Semiquantitative analysis was carried out by counting three independent microscopic fields (n = 100‐200 cells/field) for staining of specific antigen using upright microscope (Leica DM1000, Germany). Finally, the mean value was taken for each antigen to get the percentage of positive cells.

### 2.8 Patient stratification

On the basis of expression levels, patients enriched for CSC markers were grouped in four categories: CD133 (Tumor), CD44 (Tumor), CD133 (Distal) and CD44 (Distal). The enrichment was decided by comparing the expression of both markers to the median value of corresponding normal tissue samples.

### 2.9 Statistical analysis

All statistical analyses in this study were performed using GraphPad Prism 5 and SPSS version 25. For flow cytometry and immunohistochemistry data, we used un‐paired *t* test for mean comparison and *P*‐value determination between normal, tumour and distal tissues. For real‐time PCR data, one sample *t* test was performed to determine the *P*‐value comparing log_2_ fold change among tumour and distal samples. Kaplan‐Meier plots were used to determine the disease‐free survival (DFS) in followed up patients and *P*‐value was obtained by Gehan‐Breslow‐Wilcoxon test. Binary logistic regression model was used for multivariate analysis and Hosmer and Lemeshow statistical test was used to derive the *P*‐value and ODDS ratio.

## 3 RESULTS

### 3.1 Enrichment of CD133 and CD44 positive cells in colorectal tumour and distal margin

**Figure 1:**
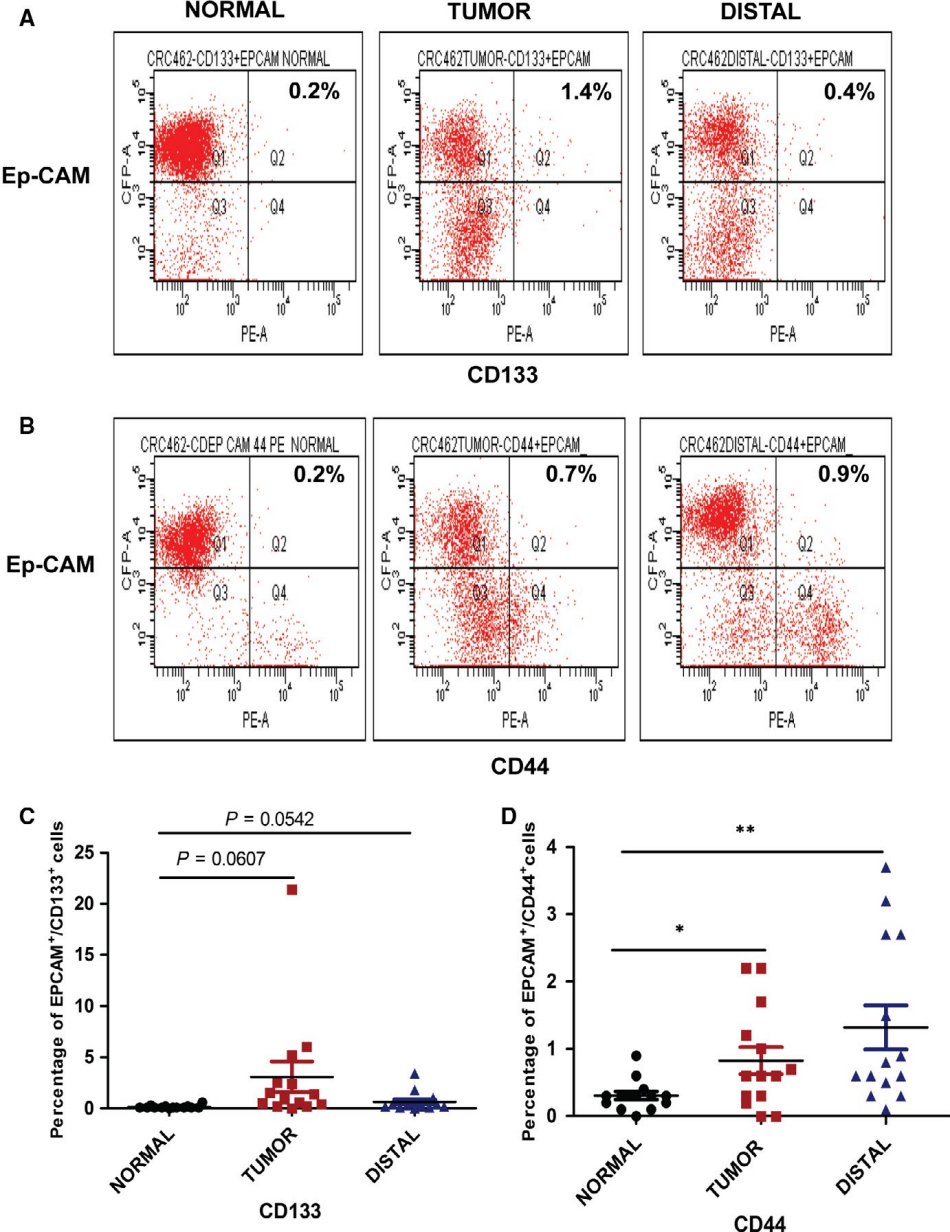
Flow cytometry analysis of CD133, CD44 and Ep‐CAM cell surface markers in normal, tumour and distal tissues. A and B, Representative quadrant plots showing percentage of double positive cells for epithelial cell adhesion molecule (Ep‐CAM) and CD133/CD44 in normal, tumour and distal tissues. C and D, Scatter plot showing quantified data of Ep‐CAM and CD133/CD44 double positive cells in all tissues. Each dot represents a sample (n = 14). Unpaired *t* test results showing *P* ≤ 0.05, ≤0.01 are represented by *, ** respectively, ns‐not significant

CD133 and CD44 antigens happen to be the most widely studied CSCs markers in CRC and combination of these markers could be helpful in identifying CSCs.^18^ In this study, we investigated CRC patients for cellular expression of CD133 and CD44 antigen using flow cytometry in normal, tumour and distal tissues obtained post surgery. To remove the immune cells in our analysis, we performed CD45 depletion using MACS beads. All the cells were stained for epithelial cell adhesion molecule (Ep‐CAM) along with either CD44 or CD133 antigen to remove non‐epithelial cells in analysis (Figure 1A,B). Results showed that mean percentage population of cells expressing both Ep‐CAM and CD44 antigens were significantly higher in tumour and marginally significant in distal margin compared to normal tissues (0.83 ± 0.20, *P* = 0.0185), and (1.32 ± 0.32, *P* = 0.0050) vs (0.31 ± 0.06) respectively. On the other hand, Ep‐CAM and CD133 expressing cells showed a very high enrichment in case of tumours (3.0 ± 1.49, *P* = 0.06) compared to the distal tissues (0.6571 ± 0.2449, *P* = 0.0542). CD133‐expressing cells were found to be very less in normal tissue with a mean of 0.1571 ± 0.03 882 (Figure 1C,D). The above results show enrichment of CD133 and CD44 positive cells in tumour as well as distal surgical margin tissues after surgery.

### 3.2 Enriched expression of CD133 and CD44 protein in tumour and pathologically negative distal margin found specific to epithelial cells

Enrichment of CD133 and CD44 positive cells in distal margin indicated the presence of CSCs and hence it was essential to understand the tissue architecture of distal margin. To this end, we performed hematoxylin and eosin staining on normal, tumour and distal tissues. Histology showed no signs of hyperplasia or disrupted tissue morphology in normal and distal tissues. Both normal and distal margin exhibited organized epithelial cells confined within intestinal crypts unlike tumour tissues (Figure S1).

**Figure 2:**
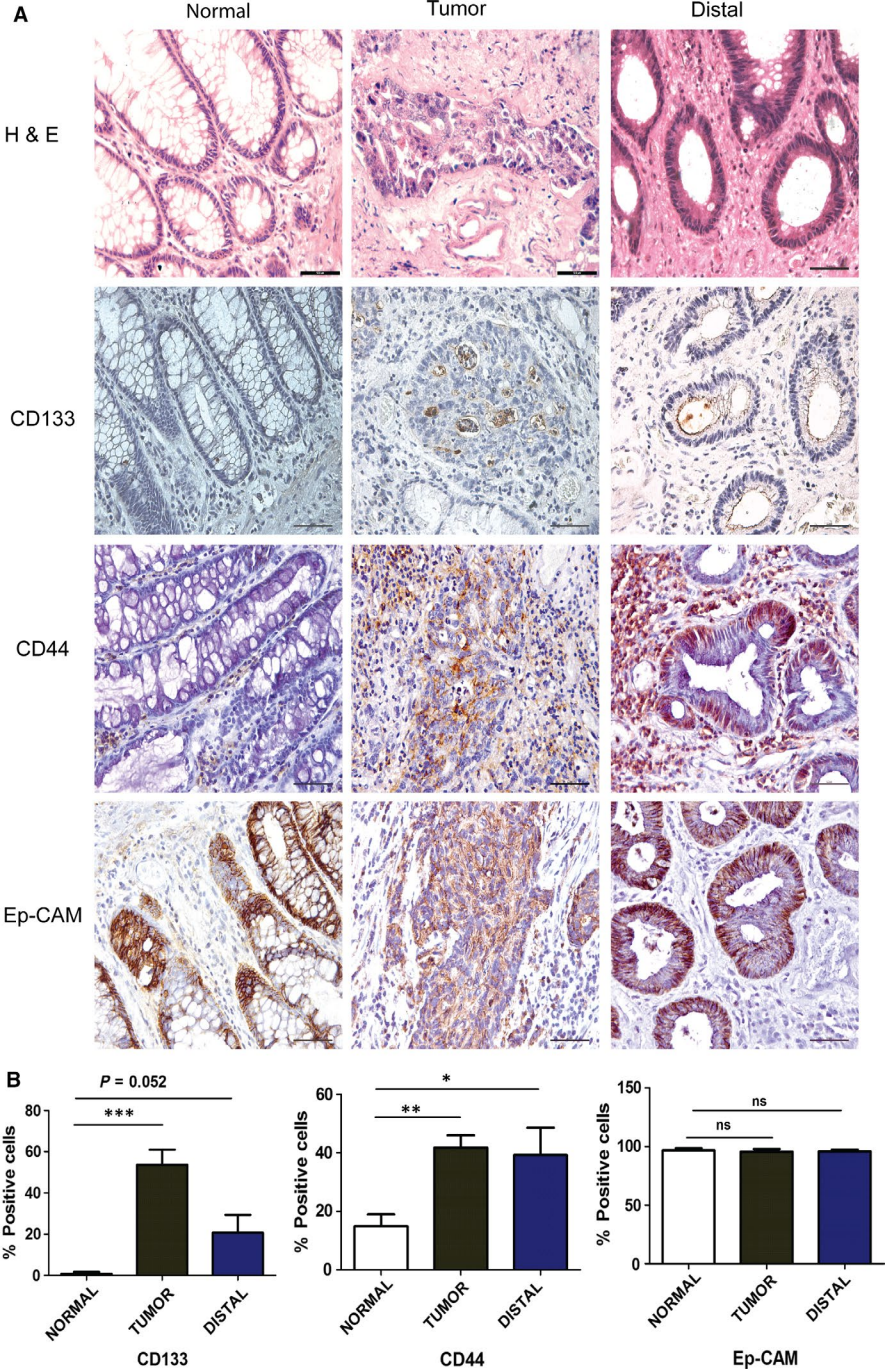
CD133, CD44 and epithelial cell adhesion molecule (Ep‐CAM) protein expression in colorectal cancer and distal margin tissues. (A) Hematoxylin and eosin staining and immunostaining of CD133, CD44 and Ep‐CAM proteins in all tissues. (B) Bar graph showing semi‐quantitative data of immunostaining for all three proteins with standard error mean and unpaired *t* test results showing *P* ≤ 0.05, ≤0.01, ≤0.001 are represented by *, **, *** respectively (n = 6), ns‐not significant. Scale bar is 12 µm

Further, we investigated the expression and localization of CD44, CD133 and Ep‐CAM proteins in all tissues using immunohistochemistry. Results showed that CD133 staining was strictly confined to the luminal surface of epithelial crypts, whereas CD44 staining was found in epithelial as well as stromal cells (Figure 2A). CD133 mean percentage positive cells were found significantly higher in tumour compared to normal (53.72 ± 7.337 vs 0.8580 ± 0.8580, *P*‐value < 0.0001). Despite having a normal histology, distal tissues showed an enrichment of the CD133% positive cells with a statistical significance closer to cut‐off value (20.68 ± 8.680, *P* = 0.0527) (Figure 2B). CD44 mean percentage positive epithelial cells were significantly higher in tumour and distal tissue compared to normal tissues (41.77 ± 4.234, *P* = 0.0017) and (39.24 ± 9.362, *P* = 0.0439) vs (14.95 ± 3.961) respectively (Figure 2B). Ep‐CAM showed higher intensity of staining in tumour and distal tissues (Figure 2A), although there was no difference in number of positive cells among the three tissues (Figure 2B). Importantly, enrichment of both the Ep‐CAM^+^/CD44^+^ and Ep‐CAM^+^/CD133^+^ cells in distal margin shows molecular alteration associated with enriched expression of putative CSC markers in margin tissues. Put together, histopathology analysis showed lack of neoplastic characteristics in normal and distal tissues but immunohistochemistry showed enriched expression of CD133 and CD44 in epithelial cells of tumour as well as distal tissues. This suggests that although distal margin shows regular tissue architecture like normal tissues, it could exhibit expression of antigens associated with CSCs.

### 3.3 Tumour and distal margin share common genetic alterations of cancer and stem cell regulatory genes along with enriched expression of β‐catenin and Oct4

**Figure 3:**
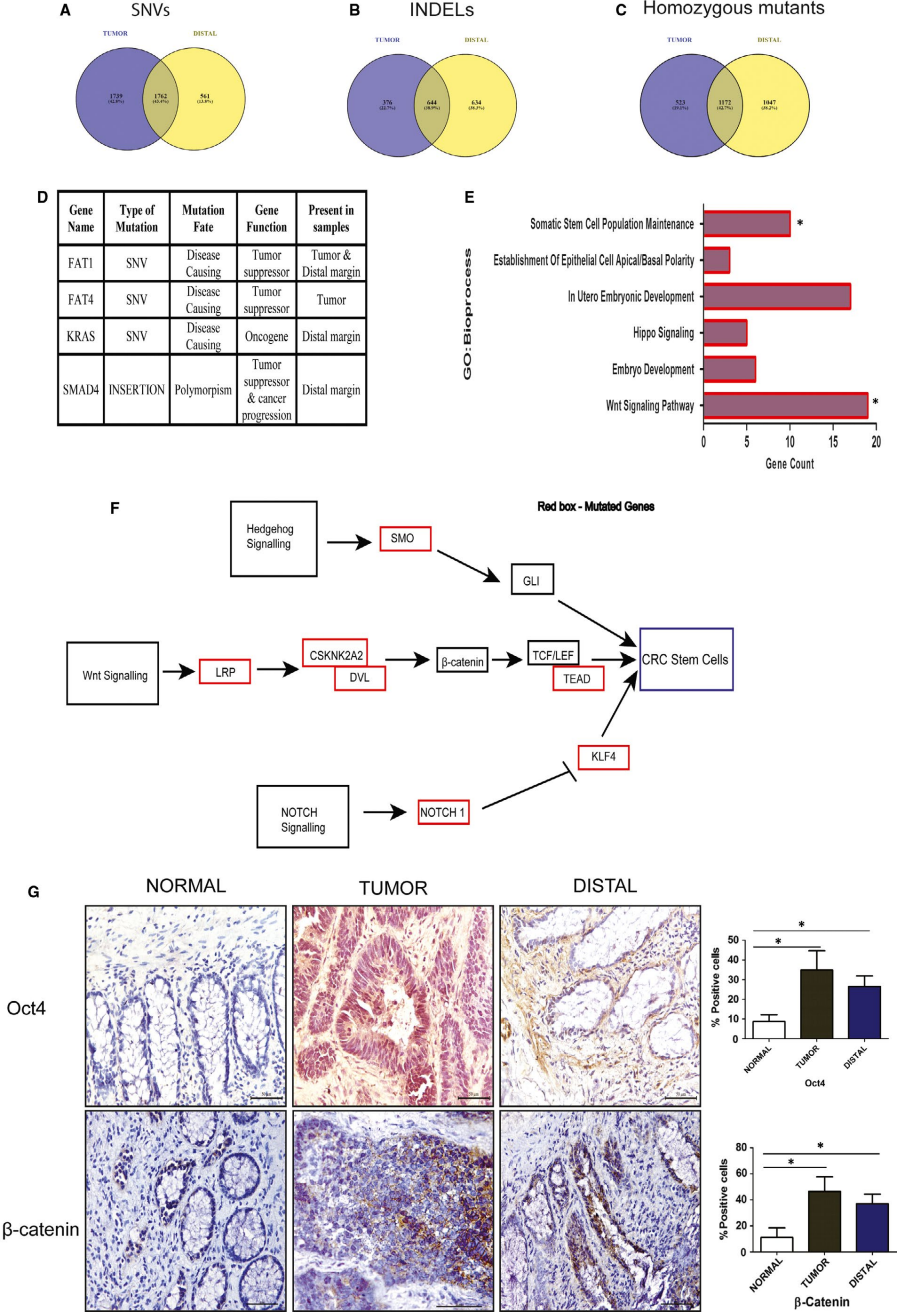
mRNA sequencing showing common genetic alterations and enrichment of Wnt signalling in CRC and distal margin tissues. A‐C, Venn diagram showing common SNVs and INDELs in distal margin and CRC. D, Table showing driver homozygous mutant genes in tumour and distal margin. E, Bar plot showing early development and stem cell maintenance bioprocess obtained from DAVID analysis of common homozygous mutation gene list. F, Schematic diagram showing crucial genes found mutated (in red box) and involved in Wnt, Notch and Hedgehog signalling in distal margin and CRC. G, Immunostaining of Oct4 and β‐catenin proteins in normal, tumour and distal margin. Bar graph showing semi‐quantitative data of immunostaining for two proteins with standard error mean. Unpaired *t* test results showing *P*‐value ≤0.05 represented by * (n=6), ns‐not significant. Scale bar is 12 µm

We performed a mRNA sequencing of normal, tumour and distal tissues to identify any common genetic alterations shared between distal margin and tumour, which could possibly explain the mechanism of enrichment of CSCs in pathologically negative distal margin. We found 1762 (43.4%) and 644 (38.9%) common single nucleotide variations (SNVs) and insertions and deletions (INDELs) respectively between tumour and distal margin (Figure 3A,B). We filtered out the heterozygous mutants from further analysis to obtain only significant mutants (SNVs & INDELs), which were found to share 42.7% (1172) between tumour and distal margin ((Figure 3C). We also mapped our data with top 20 mutated genes in CRC from COSMIC data sets and found FAT1 and FAT4 to be mutated in tumour, whereas distal margin showed the presence of FAT1, KRAS and SMAD4 mutations (Figure 3D). These mutations were further analysed for their fate using Mutation Taster tool and found to be disease causing. This observation suggests a heterogeneous driver mutation load in tumour and distal margin. Further, we sorted bioprocesses associated with early development and stem cell maintenance pathways, which could be the possible culprits for CSCs signatures in distal margin in CRC (Figure 3E) using DAVID bioinformatics tool. Finally, we observed SMO, LRP, CSKNK2A2, DVL, TEAD, NOTCH1 and KLF4 mutated genes, which are crucial in the regulation of Wnt, Notch and Hedgehog signalling pathways and thus could be the possible culprit behind the enrichment of CSCs in tumour and distal margin (Figure 3F).

In addition to that, we also looked into the expression of β‐catenin and Oct4, which are core molecules in Wnt‐mediated stem cell signalling.^19^ Oct4 showed significantly increased nuclear expression in tumour and distal tissues compared to normal tissue with a mean percentage of cells (34.91 ± 9.74, *P* = 0.0445) and (26.48 ± 5.42, *P* = 0.0329) vs (8.749 ± 3.43) respectively (Figure 3G). However, β‐catenin expression was seen in both cytoplasm and nucleus of epithelial cells and a higher expression was observed in tumour and distal margin compared to normal tissues with % positive cells (46.41 ± 11.27, *P* = 0.0256) and (37.03 ± 7.35, *P* = 0.0322) vs (11.08 ± 7.41) respectively (Figure 3G). Overexpression of Oct4 and β‐catenin proteins in tumour and distal margin further strengthened our observation on the presence of cells with stem cell signatures. Put together, these results suggest the involvement of altered Wnt signalling behind the enrichment of CD133 and CD44 in tumour as well as distal tissues.

We further analysed for mRNA levels of CD133, CD44, OCT4, SOX2 and BMI1, in tumour and distal margin tissues compared to the matched normal tissues expression using real‐time PCR (Figure S2). mRNA levels did not show any significant expression for the above‐mentioned genes unlike protein expression. However, few cases showed enrichment of CD133 and CD44 expression in distal margin tissues as well and CD44 showed a significantly enriched expression in tumours (Log_2_FC of 1.09, *P* = 0.0044) (Figure S2B).

### 3.4 Enriched CD133 expression in distal margin associated with a higher recurrence rate and lower DFS in CRC patients

**Figure 4:**
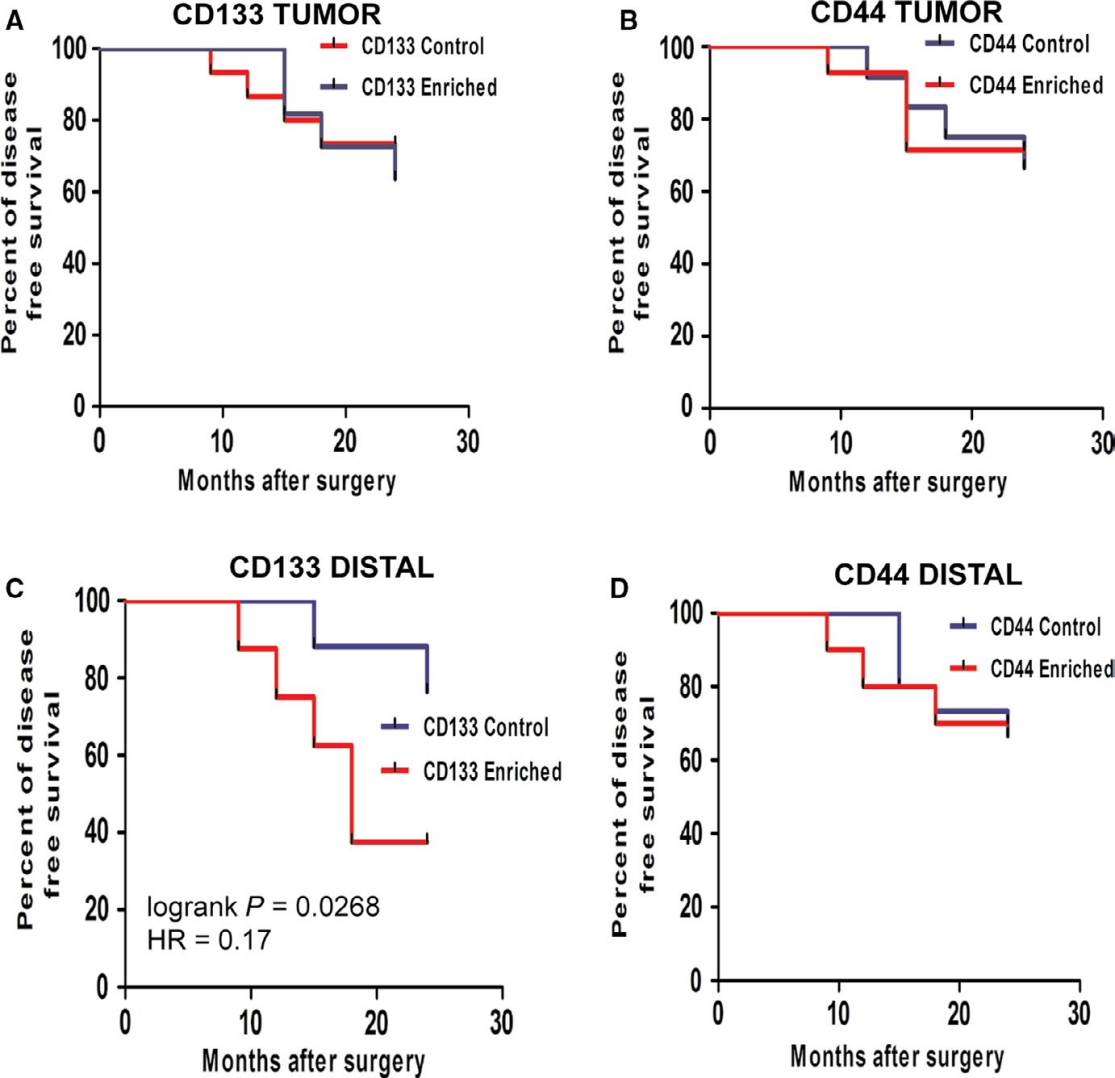
Kaplan‐Meier plot showing DFS analysis for putative CSC markers enriched group. (A and B) Plot showing tumour tissue with CD133 high (n = 15), CD133 low (n = 18) and CD44 high (n = 19) and CD44 low (14) markers respectively. (C and D) Plot showing distal margin tissue enriched for CD133 (n = 10), CD133 low (n = 23) and CD44 high (n = 14), CD44 low (n = 19) marker respectively

To investigate the clinical significance of the enrichment of CD133 and CD44 markers in CRC and distal margin tissues, all the patients quantified for CD133 and CD44 expression were followed‐up to a period of 24 months from the day of tumour excision. From follow‐up data, we observed that among all the enriched groups, CD133 (Distal) patients showed a significantly higher recurrence rate of 50% (Table 1). To delineate the DFS in the above‐mentioned groups of patients, we used Kaplan‐Meier plot. CD133 (Distal) and CD44 (Distal) showed an early recurrence with first event occurring at 9 months compared to 15 months in negative groups without any effect on overall DFS (Figure 4C,D). However, no significant difference in DFS was found among CD133 (Tumour), CD44 (Tumour) and CD44 (Distal) groups compared to control group (Figure 4A,B,D). Interestingly, patients enriched for CD133 in distal margin showed significantly lower DFS of 50% compared to CD133 control group with 82% (*P* = 0.02, HR = 0.18)(Figure 4C). This is significantly lower than other enriched groups with a DFS of more than 70%. Further, multivariate analysis results also supported the relation between high expression of CD133 in distal margin with disease recurrence with a ODDS ratio of 15.30 and *P* = 0.03 ( Table 2). However, we could not find any significant association of recurrence with status of tumour stage, lymphnode and distal margin resection length (Table 2).

**Table 1:** Showing disease recurrence percentage in CSC markers enriched in tumour and distal groups

CSC marker	Recurrence percentage
Tumour	
CD133 (4/15)	26.66
CD44 (4/19)	21.05
Distal margin	
CD133 (5/10)	50
CD44 (3/14)	28.57

**Table 2:** Multivariate analysis showing relation between CSC markers enrichment in distal margin and independent clinical prognostic factors with disease recurrence in CRC

Prognostic factors	Coefficient	SE	Wald	ODDS ratio	*P*‐value
CD133 in distal margin (High vs Low)	2.730	1.318	4.291	15.338	0.038
CD44 in distal margin (High vs Low)	−0.394	1.131	0.121	0.674	0.727
Node (Positive vs Negative)	−1.507	1.291	1.362	0.222	0.243
Tumour Stage (>T2 vs <T2)	0.687	1.130	0.370	1.988	0.543
Length of distal surgical resection margin (>3 cm vs <3 cm)	−0.080	1.045	0.006	0.923	0.939
Metastasis (Positive vs Negative)	1.576	1.582	0.993	4.837	0.319

CRC, colorectal cancer.

These results clearly suggest that CD133 expression in distal margin could have crucial role in disease recurrence in CRC.

## 4 DISCUSSION

In this study, we investigated the expression of putative CSC markers and Wnt signalling molecules associated with regulation of CSC in CRC and distal margins in patients enduring curative surgery. Surgical margins have been assessed for identification of residual cells, probably responsible for disease relapse if left untreated. Circumferential and distal margins represent important prognostic factors in CRC and are routinely checked for presence of residual cells after surgery using conventional histology.^5^, ^20^, ^21^ Previous reports suggest a relation between local recurrence and survival with resection margin status.^22^, ^23^ In some cases of patients treated with pre‐chemoradiotherapy, tumours and margins may not depict any residual cells (also known as minimal residual cancer) during pathological examination; however, these undetectable tumour cells or clusters could possibly be identified using molecular markers. A recent study in breast cancer has shown that the residual cells that persisted after chemoradiotherapy were found to be enriched in CSCs.^24^ As CSCs are thought to be the most nasty cells in a tumour, there is a possibility of their escape upon chemoradiotherapy and manifesting minimal residual disease.^25^ Recent reports have shown that CD133 marker could be a crucial prognostic factor as higher expression of CD133 was found to be associated with poor clinical outcomes in CRC.^26^ Another study has revealed an association between loco‐regional recurrence and expression of CD133 and CD44 CSC markers in rectal cancer suggesting their importance in disease prognosis.^27^ However, molecular studies on margins associated with CRC are lacking; hence we studied the tumour and its associated distal margin for identification and quantification of putative CSC markers and regulatory genes.

Ep‐CAM^+^/CD44^+^ and Ep‐CAM^+^/CD133^+^ cells happen to be the most studied CSC markers in CRC.^14^, ^18^ Higher expression of Ep‐CAM has already been reported in CRC and revealed to have high tumourigenic potential in combination with CD44 expression.^14^, ^28^, ^29^ Our flow cytometry analysis showed significant enrichment of Ep‐CAM^+^/CD44^+^ and Ep‐CAM^+^/CD133^+^ cells in distal margin after curative surgery. Interestingly, the abundance of Ep‐CAM^+^/CD44^+^ cells in distal margin and Ep‐CAM^+^/CD133^+^ cells in tumour tissues was intriguing (Figure 1B). In concordance with above findings, immunohistochemistry also showed a significant enrichment for CD133 and CD44 in tumour and distal tissues (Figure 2B). CD133 staining was found confined to luminal surface of crypts whereas CD44 stained epithelial and stromal cells (Figure 2A). However, Ep‐CAM showed no significant variation across normal, tumour and distal margin tissues, although the staining was more intense in tumour and distal margin (Figure 2A). This heterogeneity in abundance of CSCs in tumor and distal tissues could have significant clinical importance as Ep‐CAM^+^/CD44^+^ cells are invasive in nature and possess higher metastatic potential.^14^ Therefore, the presence of these cells at distal margin raises the concern for the presence of invasive cancer cells, which could lead to distant metastasis if left unidentified. On the other hand, enrichment of Ep‐CAM^+^/CD133^+^ cells are known to be therapy resistant^32^, ^33^ and abundance of these cells in tumour and distal margin suggests the presence of drug‐resistant cells.

Interestingly, the enrichment of CD133 and CD44 positive cells were not evident in all cases of patients sample analysed (Table S4), showing inter‐tumour heterogeneity among CRC patients. Enriched expression of CD44 and CD133 in tumour and distal margin could suggest the presence of disseminated cells from the tumour (residual cells) or may be due to the molecular alteration caused by treatment given. A recent study has reported that the expression of CSC markers after preoperative chemoradiotherapy has independent role in predicting recurrence‐free survival in rectal patients.^34^ Yet, most importantly the question which needs to be addressed happens to be the causal reason behind this heterogeneity in CSC abundance in tumour and distal margin upon therapy and their role in disease prognosis.

To explore the mechanism of CSCs enrichment in pathologically negative distal margin, we performed RNA sequencing of tumour and distal tissue keeping normal tissue as reference. This study revealed a significant number of cancer‐associated genes, which were found to be mutated in both tumour and distal margins (Figure 3A‐D). This finding suggests that histopathologically normal cells in distal tissue possess genetic alterations similar to tumour. In addition, a significant number of mutated genes were found to be involved in cell proliferation, migration, drug response, adhesions, ECM organizations needed for cancer cell growth and invasion. Importantly, we found Wnt signalling genes to be enriched among the top 20 bioprocess in our analysis (Figure S4). Enrichment of mutated genes associated with stem cell maintenance bioprocess in both tumour as well as distal margin supports our observation on presence of CSCs in distal margin (Figure 3E). However, exposure to chemoradiotherapy has been already studied to induce stemness characteristics in various cancers. So it is obvious that it can affect normal surrounding tissues of tumour as well. Interestingly, RNA sequencing study revealed distinct genetic differences between normal tissue compared to distal and tumour. This further shows that CSC signatures in distal margin cannot be solely due to chemoradiotherapy and distal adjacent tissue could share a tumour field effect unlike normal tissue in CRC. Among altered CSC maintaining genes, we observed a significant number of Wnt signalling components (Figure 3E). Studies have shown the regulatory role of Wnt signalling in the expression of CD133 and CD44 in CRC.^35^, ^36^ Oct4 and β‐catenin are the two major Wnt pathway genes which are involved in stem cell maintenances.^37^ In our IHC studies, we observed an increased expression of Oct4 and β‐catenin in tumour and distal margins, whereas normal tissues showed minimal expression (Figure 3G). Higher Oct4 and β‐catenin expression thus again supports the enrichment of CSC in tumour and distal margin tissues. In addition to that, these observation implies that the active involvement of Wnt signalling could be the possible driver behind the enrichment of CD133 and CD44 markers. Real‐time q‐PCR analysis of CRC tissues did not show significant enrichment of CSCs markers in case of tumour and distal margin like immunophenotyping (Figure S2). This could be due to limitation of real‐time PCR in specificity as involvement of stromal cells could influence the results.

The expression of CD133 in CRC has already been reported to be involved with drug resistance and disease recurrence and also associated with lower patients survival.^15^, ^32^, ^33^, ^38^ However, our follow‐up study showed higher recurrence rate in group with CD133 enrichment in distal margin compared to CD133 tumour enriched group (Table 1). Surprisingly, 50% patients enriched for CD133 in distal margin recurred, whereas rest of the groups showed recurrence rate of less than 30% (Table 1). In addition to that, Kaplan‐Meier plot showed significantly lower DFS for CD133 enriched group in distal margin compared to control group as well as CD44‐ and CD133 enriched group of tumour (Figure 4). Obtaining an adequate length of distal resection margin in colon cancer is relatively easier compared to rectal cancer, as the presence of bony pelvis and sphincter muscle makes the task difficult. Moreover, rectal cancer cases are treated with preoperative chemoradiotherapy. Thus, we analysed the DFS in only rectal cancer cases as it would be interesting to know the relation between CSC enrichment in distal margin and recurrence in rectal cancer. Kaplan‐Meier plot revealed a significantly lower DFS (28%, *P* = 0.0007) in CD133‐enriched distal group compared to other CSC markers enriched groups in tumour and distal tissues (Figure S3). These results further strengthen the functional role of CSCs in distal surgical margin in CRC prognosis. Recent reports in CRC advocate use of molecular analysis for better prediction of disease recurrence as clincopathological parameters such as tumour stage, lymphnode positivity may prove insufficient for the same.^39^, ^40^ Our multivariate analysis resulted in a similar observation, as we could not find any significant association of disease recurrence with any clinicopathological parameters (Table 2). Interestingly, low distal resection length also did not show any significant relation with recurrence. However, higher expression of CD133 in distal margin found to have significant association with recurrence (*P* = 0.03), but CD44 expression did not show similar result (*P* = 0.82) (Table 2). Altogether these results advocate the potential of CD133 marker in CRC prognosis. We further propose that CD133 expression in distal margin should be evaluated in a larger cohort of sample to validate its potential role as an independent prognostic marker in CRC.

As distal resection margin length remains a crucial parameter in cases of rectal cancer due to challenges associated with sphincter preserving surgery,^20^, ^41^ our study suggests molecular evaluation of distal margins along with histopathology for better prognostication of patients with CRC.

## 5 CONCLUSION

In conclusion, our study for the first time showed that in CRC, distal margin harbouring cells with CSCs characteristics can affect the patient's prognosis. In addition, enrichment of CD133 and CD44 in distal margin are independent of its pathological status. Although we accept the limitation in our study, for lacking a larger cohort, we further propose that CD133 expression status in distal margin could be a crucial factor in disease progression in CRC patients and could be used as a prognostic marker. As residual cells could be the sole cause behind local recurrence and metastasis, and histological analysis alone may not provide adequate information regarding the heterogeneity of tumour cell types present or molecular alternation set in the tumour and its margins. Based on these observations, we propose that molecular assessment of margin in CRC could be an added tool supplementing histopathology for better treatment and management of CRC patients in the long run.

## ETHICAL APPROVAL AND CONSENT TO PARTICIPATE

All human tissue samples were obtained from Regional Cancer Centre, Trivandrum, Kerala, India after approval of the Regional Cancer Centre Ethical committee (HEC No. 43/2011) and donors that signed written informed consent.

## CONFLICT OF INTEREST

The authors declare no conflict of interest.

## AUTHORS’ CONTRIBUTIONS

TP, KP, MP conceived, planned and carried out the experiments, lead in data analysis and manuscript writing. KC performed the surgery and helped in obtaining the samples and their storage. AN conceived, designed the project and supervised its execution, manuscript proofreading and provided critical feedbacks in manuscript writing. All authors provided critical feedback and helped shape the research, analysis and manuscript.
