# Supplementary figures and images for: Augmented CD133 expression in distal margin correlates with poor prognosis in colorectal cancer
Source: J Cell Mol Med. 2019 Apr 4;23(6):3984–94. doi: 10.1111/jcmm.14284 (PMC6533563; doi:10.1111/jcmm.14284)

**Supplementary figure 1.**


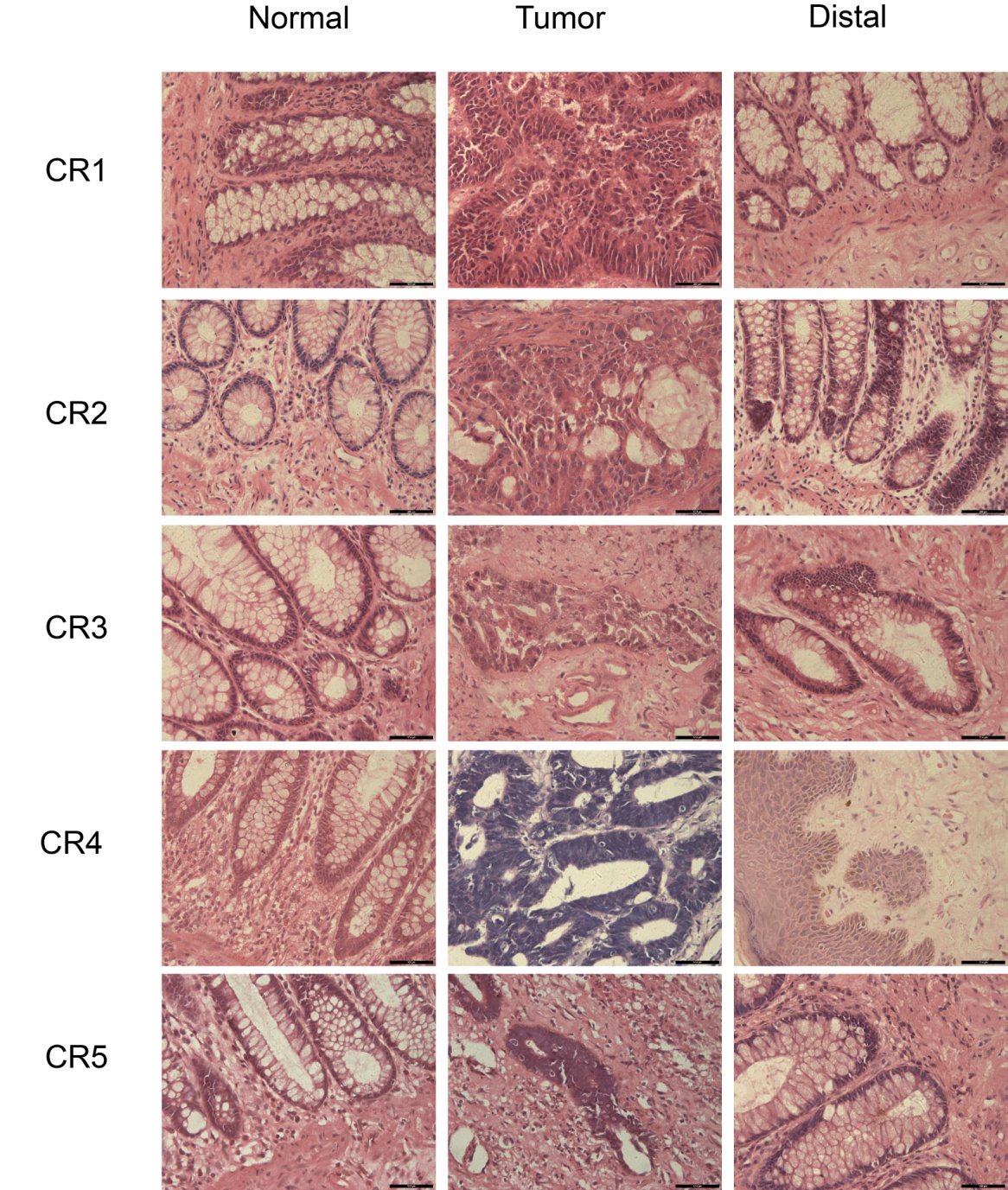


**Supplementary figure 2.**


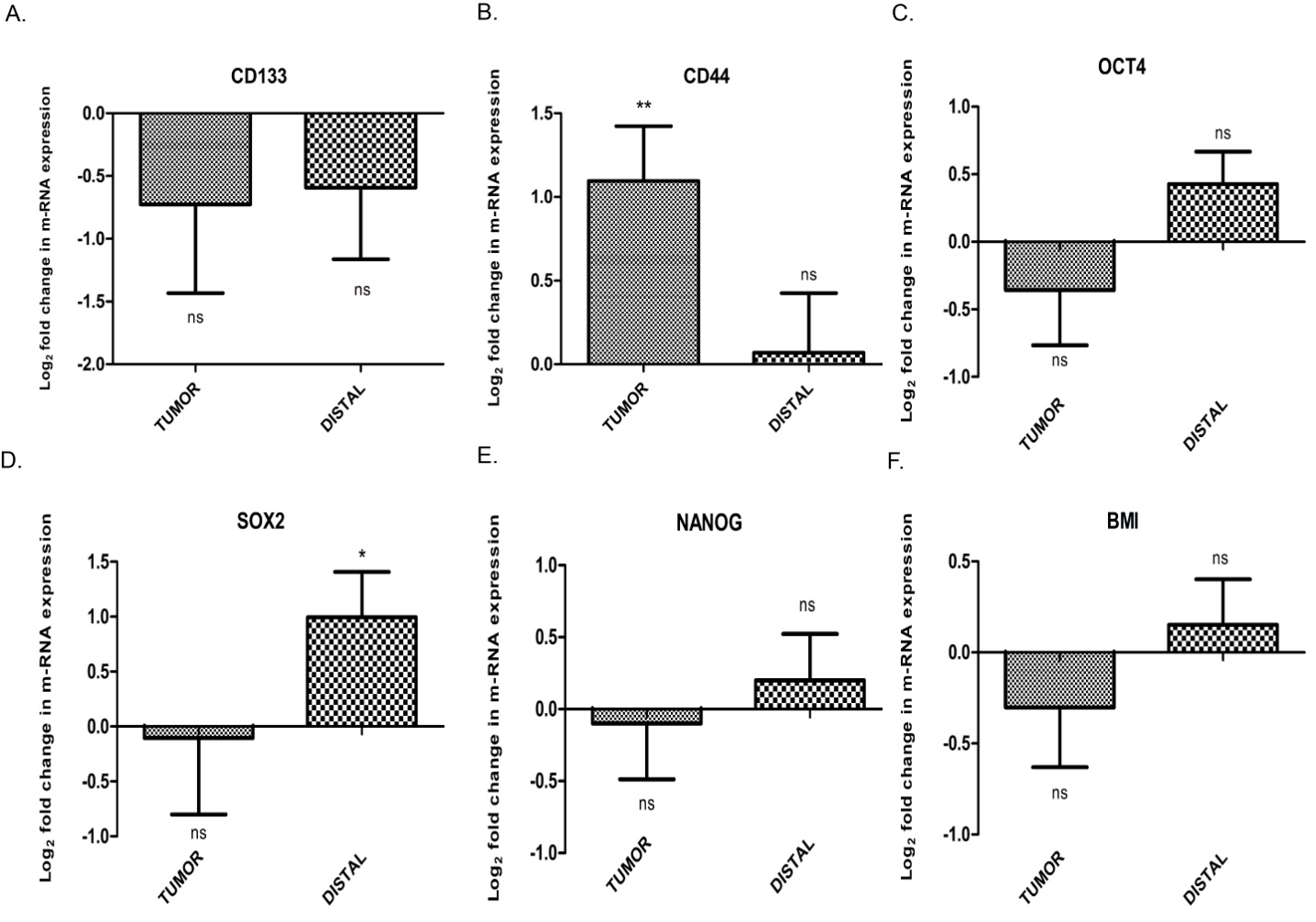


**Supplementary Figure 3.**

**
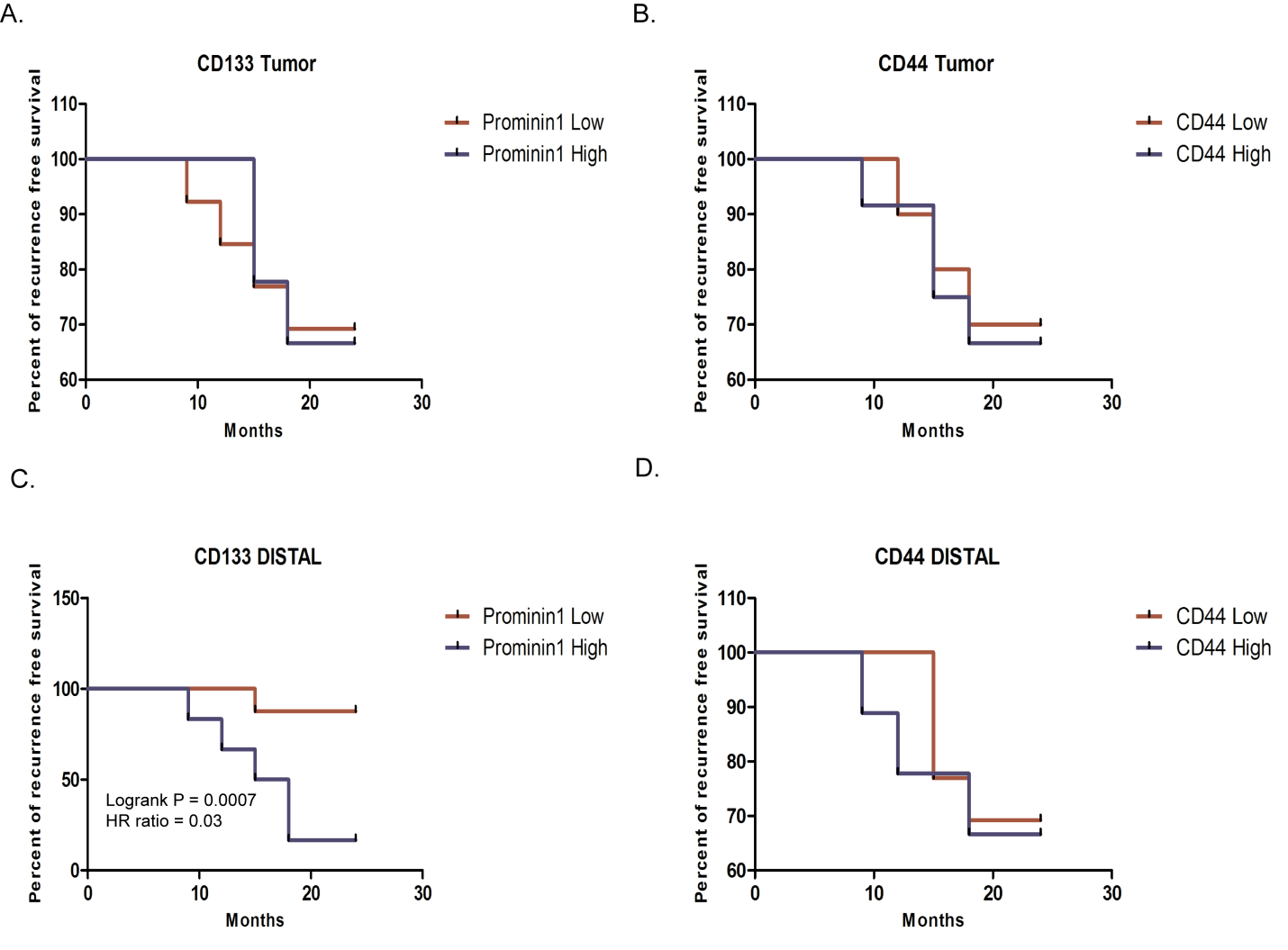
**

Supplement: Supplementary file 1 [file JCMM-23-3984-s001.docx]
