# Supplementary material for: Augmented CD133 expression in distal margin correlates with poor prognosis in colorectal cancer
Source: J Cell Mol Med. 2019 Apr 4;23(6):3984–94. doi: 10.1111/jcmm.14284 (PMC6533563; doi:10.1111/jcmm.14284)
Supplement: Supplementary file 2 [file JCMM-23-3984-s002.docx]

**Supplementary Table 1.**

A total of 33 colorectal cancer patient’s biopsies were used in this study. Details of the patient’s demographics are given in this table.

| **Patients characteristics** | | | | |  |
| --- | --- | --- | --- | --- | --- |
|  |  |  |  |  |  |
| **Total number of patients** | |  | | 33 |  |
| **Median Age** | |  | | 59 Years |  |
| **Sex** | |  | |  |  |
|  | | Women | | 12 |  |
|  | | Men | | 21 |  |
| **Depth of Invasion** | |  | |  |  |
|  | | ≥p T2 | | 24 |  |
|  | | ≤p T2 | | 9 |  |
| **Lymphatic Invasion** | |  | | 2 |  |
| **Lymph node metastasis** | |  | |  |  |
|  | | Negative | | 13 |  |
|  | | Positive | | 20 |  |
| **Distant metastsis** | |  | |  |  |
|  | | Negative | | 29 |  |
|  | | Positive | | 4 |  |
| **Histologic type** | |  | |  |  |
|  | | Well to moderately differentiated | | 32 |  |
|  | | Poorly differentiated | | 1 |  |
| **Site of tumor** | |  | |  |  |
|  | | Colon | | 10 |  |
|  | | Rectum | | 23 |  |
| **Distal margin** | |  | |  |  |
|  | | Positive | | 0 |  |
|  | | Negative | | 33 |  |
| **Proximal margin** | |  | |  |  |
|  | | Positive | | 0 |  |
|  | | Negative | | 33 |  |
|  | |  | |  |  |
|  |  | |  | | |

**Supplementary Table 2.**

A total of nine genes were evaluated for gene expression using real-time q-PCR in normal, tumor and distal biopsies. Details of both forward and reverse primers sequences are given in this table.

| **Sr.No.** | **Genes Name** | **Primer Sequence 5' to 3' -** |
| --- | --- | --- |
| 1 | \| OCT4-F \|  \| \| --- \| --- \| | GGGAGATTGATAACTGGTGTGTT |
| 2 | OCT4-R | GTGTATATCCCAGGGTGATCCTC |
| 3 | SOX2-F | AGGATAAGTACACGCTGCCC |
| 4 | SOX2-R | TAACTGTCCATGCGCTGGTT |
| 5 | BMI-F | AGTATGAGAGGCAGAGATCGGG |
| 6 | BMI-R | CTGCTAAGCCGGAACGGG |
| 7 | TWIST1-F | CCTCCAAGTCTGCAGCTCTC |
| 8 | TWIST1-R | AAAAAGAAAGCGCCCAACGG |
| 9 | NANOG-F | CAGAAGGCCTCAGCACCTAC |
| 10 | NANOG-R | ATTGGAAGGTTCCCAGTCGG |
| 11 | EPCAM-F | GCTGGCCGTAAACTGCTTTG |
| 12 | EPCAM-R | ACATTTGGCAGCCAGCTTTG |
| 13 | CD133-F | GCATTGGCATCTTCTATGGTT |
| 14 | CD133-R | CGCCTTGTCCTTGGTAGTGT |
| 15 | CD44-F | AGAAGGTGTGGGCAGAAGAA |
| 16 | CD44-R | AAATGCACCATTTCCTGAGA |
| 17 | L19 -F | GCGGAGAGGGTACAGCCAAT |
| 18 | L19 -R | GCAGCCGGCGCAAA |

**Supplementary Table 3.**

Table shows the details of antibodies used in Flow cytometry and Immunohistochemsitry.

| **Antibody Name** | **Company name and Catalog No.** |
| --- | --- |
| Anti-Ep-CAM | Abcam,ab 71916, USA |
| Anti-CD44 | Abcam,ab157107, USA |
| Anti-OCT4 | Abcam,ab18976, USA |
| Anti-β Catenin | CST,8480S, USA |
| Anti-CD133 | CST,#86781,USA |
| CD133 (AC133)-PE | Miltenyi,130-098-826,Germany |
| CD44-FITC | Miltenyi, 130-098-210,Germany |
| CD326-VioBlue | Miltenyi, 130-098-092,Germany |

**Supplementary Table 4.**

Table showing the percentage of CRC patients enriched for CD133 and CD44 markers by quantitative analysis using Flow cytometry , Real-time q-PCR and Immunohistochemistry.

|  | **Numbers** | **Percentage** |
| --- | --- | --- |
| Total analyzed CRC samples | 33 | 100 |
| Number of tumor samples enriched for CD133 | 15 | 45.4 |
| Number of tumor samples enriched for CD44 | 19 | 57.5 |
| Number of distal samples enriched for CD133 | 10 | 30.3 |
| Number of distal samples enriched for CD44 | 14 | 42.4 |
